# Smartphone and email capabilities of heart failure patients

**DOI:** 10.1093/ehjdh/ztab008

**Published:** 2021-01-29

**Authors:** Gayle Campbell, Vivian Auyeung, Tevfik F Ismail

**Affiliations:** 1 Cardiology Department, Guy’s and St Thomas’ NHS Foundation Trust, London SE1 7EH, UK; 2 School of BMEIS, King’s College London, London, UK; 3 Institute of Pharmaceutical Science, King’s College London, London, UK

In 2008, the Apple app store was launched and paved the way for mobile apps to be easily accessible on a global scale. The number of global smartphone users is thought to be 3.5 billion^1^ and in 2017, there were 325 000 health apps available.^2^ There is growing interest in using apps to support the management of long-term conditions, such as heart failure.

Heart failure is a complex clinical syndrome.^3^ Monitoring of symptoms and adherence to medications is essential to prevent decompensation. However, wide variability in medication adherence in heart failure patients has been shown (40–60%),^4^ which prevents the full benefits of these life-saving medications being realized and potentially limits other available interventions.

As healthcare providers, being able to offer information in differing formats is essential to try and meet the needs of the diverse population. The complexity of heart failure patients means that multiple methods are often needed to ensure patients have the necessary information to empower them to be involved in the management of their condition.

However, interventions delivered via apps are often criticized for being limited to a younger audience, particularly for heart failure patients where the average age of diagnosis in the UK is 77.^5^ We, therefore, conducted a survey looking at the digital capabilities of 50 heart failure patients with both reduced ejection fraction and preserved ejection fraction who were either admitted to hospital or seen in an outpatient clinic.

Fifty patients completed a digital usage questionnaire during the Autumn of 2020 (mode age 70, 9 female). The first questions related to mobile phone ownership (*Table 1*), with 96% (*n* = 48) of patients stating they owned a mobile phone. The most frequently listed duration for owning their current device was less than a year, 29% (*n* = 14), with another 23% (*n* = 11) having owned their device for over 5 years. This variation in device age could impact specific app functionalities if older operating systems are in use. Of those that owned a phone, 87% (*n* = 42) stated that their mobile was a smartphone, i.e. they are able to download mobile applications. Patients without a phone or smartphone were not asked if they had a relative who supported them that did have access to this technology. Of the 42 with smartphones, 52% (*n* = 22) had an android device and 40% (*n* = 17) had an iPhone with 7% (*n* = 3) listing their operating platform as ‘other’. This highlights that any new app development needs to be available on both iOS and Android.

**Table 1:** Digital capabilities by patient age

Age	Do you own a mobile phone? Yes (%)	If yes, is it a smartphone Yes (%)	Do you have internet at home? Yes (%)	Do you have an email address? Yes (%)
<40 (*n* = 3)	3 (100%)	3 (100%)	3 (100%)	3 (100%)
40–49 (*n* = 3)	3 (100%)	3 (100%)	3 (100%)	3 (100%)
50–59 (*n* = 11)	11 (100%)	11 (100%)	11 (100%)	9 (91%)
60–69 (*n* = 10)	11 (100%)	10 (91%)	9 (82%)	9 (82%)
70–79 (*n* = 12)	15 (100%)	12 (80%)	14 (93%)	13 (87%)
80–89 (*n* = 2)	4 (50%)	2 (50%)	2 (33%)	1 (17%)
90+ (*n* = 11)	1 (100%)	1 (100%)	1 (100%)	0 (0%)

**Figure 1:**
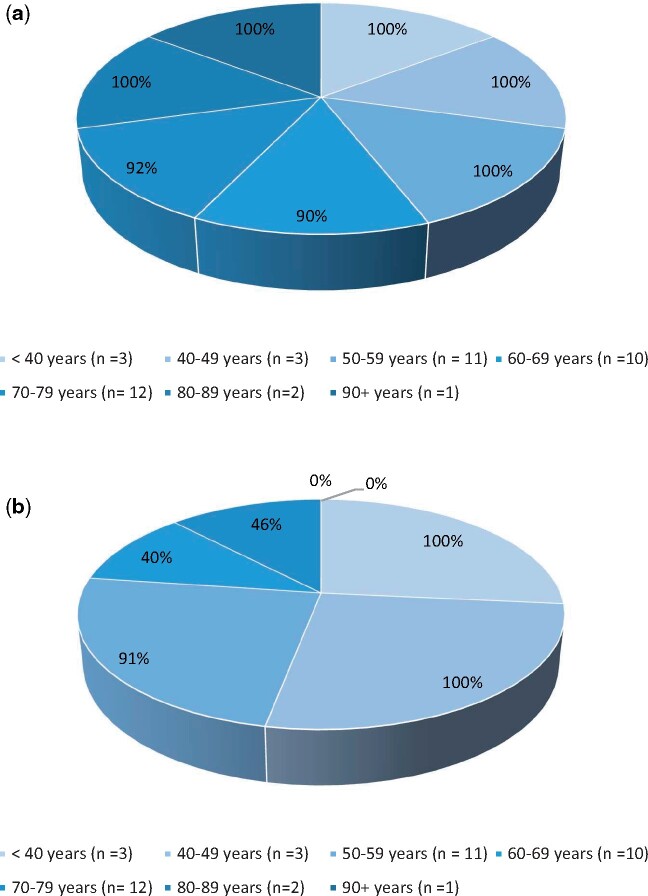
(*A*) Percentage of heart failure patients per age group that use their smartphone once a day or more. (*B*) Percentage of heart failure patients per age group that are confident or very confident with using smartphone apps.

*Figure 1A* shows that across the age groups, patients are frequently using their phones with 83% (*n* = 40) of phone owners stating that they use their phone either daily or several times a day. This high usage opens up the possibility of undertaking disease management with a smartphone-based app. However, the limiting issue to an app being utilized successfully is the confidence in the usage of apps. The results demonstrate the reducing level of confidence as the age groups increase (*Figure 1B*).

Most patients (*Table 1*) have internet at home 84% (*n* = 42), with 79% (*n* = 33) reporting their internet data are unlimited. Interestingly, the responses show that fewer patients have an email account than a smartphone, 78% (*n* = 39). This is perhaps not accurate, given that as part of the set-up with a smartphone, an email address is usually required. However, it may be that this process is utilized but that specific email account is then not used. Of those that reported having an email account, 85% (*n* = 33) reported using their email daily, 8% (*n* = 3) weekly, and the remaining split equally between monthly, yearly, and never. The majority of those with an email account are utilizing their smartphone as their primary method to use their account; 74% (*n* = 29) and 23% (*n* = 9) using a computer or tablet.

Medication adherence can be defined as either intentional or unintentional.^6^ Unintentional non-adherence is used to describe patients lack the capacity, capability, or resource to take their medicines as prescribed.^7^ An example of this would be forgetting. For smartphone users, setting an alarm can be a simple yet effective method to avoid this. However, of the phone users, only 14% (*n* = 7) used their phone to as tool to aid adherence, with the majority using the phone alarm and just one used a health app.

This survey has shown that heart failure patients of all ages have access to technology that could allow them to use a digital apps as part of the management of their long-term condition. However, when considering app development in this patient cohort, simplicity of use is fundamental given the lack of confidence in app usage, especially in older patients. Any implementation of new digital applications should involve clear user instructions or for teams to provide accessible training to patients identified as being not very confident or requiring help. These steps would go some way to ensuring that the mobile application is suitable for all and those who are willing to use this alongside standard care are provided with the opportunity to do so.

**Conflict of interest:** none declared.
